# Corrosion Products Formed on MgZr Alloy Embedded in Geopolymer Used as Conditioning Matrix for Nuclear Waste—A Proposition of Interconnected Processes

**DOI:** 10.3390/ma14082017

**Published:** 2021-04-16

**Authors:** Rémi Boubon, Jaysen Nelayah, Samuel Tardif, Xavier Deschanels, Diane Rébiscoul

**Affiliations:** 1 ICSM, CEA, CNRS, ENSCM, Université Montpellier, Marcoule, 30207 Bagnols-sur-Cèze, France; remi.boubon@cea.fr (R.B.); xavier.deschanels@cea.fr (X.D.); 2 Laboratoire Matériaux et Phénomènes Quantiques, Université de Paris, CNRS, 75013 Paris, France; jaysen.nelayah@u-paris.fr; 3 CEA, IRIG-MEM, Université Grenoble Alpes, F-38000 Grenoble, France; samuel.tardif@cea.fr

**Keywords:** magnesium corrosion, geopolymer, corrosion products, corrosion inhibitor, interconnected processes

## Abstract

Geopolymer has been selected as a hydraulic mineral binder for the immobilization of MgZr fuel cladding coming from the dismantling of French Uranium Natural Graphite Gas reactor dedicated to a geological disposal. In this context, the corrosion processes and the nature of the corrosion products formed on MgZr alloy in a geopolymer matrix with and without the corrosion inhibitor NaF have been determined using a multiscale approach combining in situ Grazing Incidence hard X-ray Diffraction, Raman microspectroscopy, Scanning and Transmission Electron Microscopies coupled to Energy Dispersive X-ray Spectroscopy. The composition, the morphology, and the porous texture of the corrosion products were characterized, and the effect of the corrosion inhibitor NaF was evidenced. The results highlighted the formation of Mg(OH)_2−x_F_x_. In addition, in presence of NaF, NaMgF_3_ forms leading to a decrease of the thickness and the porosity of the corrosion products layer. Moreover, a precipitation of magnesium silicates within the porosity of the geopolymer was evidenced. Finally, we propose a detailed set of interconnected processes occurring during the MgZr corrosion in the geopolymer.

## 1. Introduction

In France, the dismantling of French Uranium Natural Graphite Gas reactor [1] (UNGG) resulted in MgZr alloy fuel cladding waste containing uranium and fission product residues. Regarding the presence of these residues, MgZr fuel cladding is considered as an intermediate-level radioactive waste meant for geological disposal. Before their disposal, these waste materials have to be immobilized in a conditioning matrix, e.g., a hydraulic mineral binder.

Magnesium alloy is reactive regarding its corrosion in a hydraulic binder, leading to hazardous hydrogen production detrimental regarding the disposal safety. To limit hydrogen release, a geopolymer matrix has been selected as a potential mineral binder. A geopolymer is a nanoporous aluminosilicate filled with a basic poral solution (pH = 12) consisting of an alkaline solution and mainly hydrolyzed silica species [2]. This inorganic material is formed by a geopolymerization process corresponding to dissolution-recondensation reactions of dissolved species followed by inorganic polycondensation reactions [3]. This matrix presents interesting mechanical and chemical properties and has been largely studied for radioelement immobilization [4,5,6,7] such as ^133^Cs [8] and ^90^Sr [9], and the stabilization of radioactive liquid oily waste [10].

In geopolymer, when the basic poral solution is in contact with MgZr, Brucite Mg(OH)_2_ is formed and limits the alloy corrosion [11,12]. Moreover, corrosion inhibitor additives can be considered, such as NaF that favors the formation of fluorine phases, e.g., Mg(OH)_2−x_F_x_, MgF_2_, Na(K)MgF_3_, as proposed or characterized in [13,14,15,16,17], and that not modifies the geopolymer properties. These fluorine phases also limit the MgZr corrosion and thus the hydrogen production [18,19,20]. Recently, using electrochemical experiments, the corrosion of MgZr alloy embedded within the geopolymer with various NaF contents have shown that the corrosion rate and the nature of the fluorine phases formed are dependent of NaF concentration within geopolymer [21]. This result is in line with our recent experiments of natural MgZr alloy corrosion in poral solutions extracted from geopolymers with and without NaF [17]. The identification of the corrosion products (CP) formed has highlighted that the amounts of fluoride species and dissolved silica in the solution set the nature of the CP, i.e., Brucite, fluorine phases, and magnesium silicates, and probably their protective properties regarding the MgZr corrosion. However, the origin of these protective properties probably in relation with the CP porous texture have not been investigated. Moreover, in our experiments, magnesium silicates precipitation was observed within the CP layer contrary to the electrochemical experiments of Barros et al. [21].

Thus, the aim of this work is to study the nature, the porous texture, and the location of the CP formed during the corrosion of the MgZr alloy embedded in a geopolymer, with or without corrosion inhibitor NaF, and the underlying processes of their formation. To reach this goal, we used a multiscale approach combining in situ Grazing Incidence hard X-ray Diffraction (GI-XRD), Raman microspectroscopy, Scanning and Transmission Electron Microscopies coupled to Energy Dispersive X-ray Spectroscopy (SEM-EDX and STEM-EDX).

## 2. Materials and Methods

### 2.1. Materials

Magnesium alloys (Magnesium: 99.5% and Zirconium 0.5 wt%, referred as MgZr) ingots of 100 × 50 × 30 mm^3^ supplied by Neyco Society (Vanves, France) were used in this study. The impurities present in the alloy are displayed in Table 1. This composition is close to the one used in the UNGG cladding.

The MgZr substrates were cut from the ingots using a diamond wire saw of 150 µm at 0.8 rpm under ethanol as lubricant. Polishing steps were performed using SiC paper of different grades (500 then 1200) using diamond suspensions of 9 and 3 µm on clothes (MD-Largo and MD-Dac) with lubricant and ethanol. Final polishing was achieved using vibrational polishing with a 40 nm colloidal silica solution mixed with 50 vol.% of ethanol on MD-NAP (Struers, France). Afterwards, the MgZr samples were cleaned in ethanol for 15 min in an ultrasonic bath, rinsed with ethanol, dried under Ar, and saved in a N_2_ glove box to avoid potential oxidation before embedding in the geopolymer.

The geopolymers 1Na_2_O–3.96SiO_2_–1Al_2_O_3_–12.5H_2_O with or without NaF, GP, and NaF-GP, respectively, were prepared as reported in our previous study [17]. This composition corresponds to the model geopolymer used as a reference in most of scientific studies [2,19,22] and for MgZr encapsulation. The activation solution prepared by the dissolution of 16.92 g of sodium hydroxide (NaOH pellets, Sigma-Aldrich, Molsheim, France, purity 99.9%,) in 130.04 g of commercial sodium silicate solution, the Betol 39T (Woellner, Germany, composed of 27.80 wt% of SiO_2_, 8.30 wt% of Na_2_O, and 63.90 wt% of H_2_O) was mixed with 3.68 mL of ultrapure water under magnetic stirring during one hour. Due to the exothermic reaction of silicate dissolution, the activation solution was cooled down to room temperature during the mixing. Afterwards, 102.44 g of metakaolin (Argical-M-1000 from AGS Mineraux, Clérac, France, composed of 54.40 wt% of SiO_2_, 38.40 wt% of Al_2_O_3_, and 7.2 wt% of impurities) was added to the activation solution and stirred during 10 min until the solution homogenization. NaF-GP was prepared by a simple addition of sodium fluoride (NaF, 99%, Strems Materials Bischheim, France) solution at 1.25 M (0.012 wt%) to the activation solution and the solution was stirred during 1 h before the metakaolin addition. For information, the composition of the poral solutions extracted from these geopolymers also reported in [17] are presented in Table 2.

### 2.2. Sample Preparation

**Figure 1:**
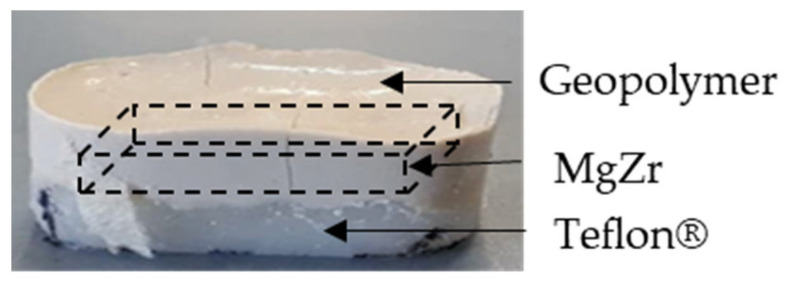
Description of a sample.

The MgZr substrates were placed in polytetrafluoroethylene holders and covered with geopolymer just after its preparation, when the geopolymer was still viscous. The two sets were prepared, including or excluding the NaF additive, and are referred hereafter as GP-NaF and GP, respectively. The samples were then placed in a desiccator containing a saturated solution of KCl, fixing a relative humidity (RH) of 82% at 25 °C [23] during 90 days to avoid the drying of the geopolymer. A typical sample is shown in Figure 1.

After the in situ characterization described below, the two samples were frozen and freeze-dried during 24 h with a LABCONO FreeZone 2.5 Freeze Dry System (Kansas, Mo, USA). This method preserves the material porosity and minimizes the crack [24]. Afterwards, cross-sections and thin foils (about 200 nm thick) of the geopolymer/MgZr samples were prepared.

### 2.3. Characterization

Samples GP and GP-NaF were characterized using Grazing Incidence hard X-Ray Diffraction (GI-XRD) at 27 keV (λ = 0.4592 Å) on BM32 beamline at the European Synchrotron Radiation Facilities (Grenoble, France). Hard X-rays were required to cross the geopolymer, and then to probe the CP formed at the surface of MgZr. To analyze various depths in the MgZr substrate, the incident angle of the beam was fixed at α_i_ = 0.1 and 0.5°. In a first approximation, considering that the surface mainly consists in Mg(OH)_2_ and Mg, the penetration depth was assessed between 10 µm at 0.1° and a few ten of microns at 0.5° (see Figure S1). Data were acquired between 1 and 31° during 25 min with a 0.01° step. For easy understanding, GI-XRD patterns are presented as a function of d-spacing (Å)$\;(d=\frac{\lambda }{2\sin\theta }$).

Raman microspectroscopy was used to characterize CP layer from samples prepared as cross-sections. Raman measurements were carried out in backscattering geometry configuration, on a Horiba Jobin Yvon LabRAM Aramis confocal Raman microscope (France) at the Institut de Chimie Séparative (Marcoule, France), using an excitation wavelength of λ = 532 nm with a laser spot size of about 1 mm with an objective of ×100 working distance. The analysis was performed by the embedding resin, which results in several peaks between 1000 and 3400 cm^−1^. Thus, Raman data were recorded between 3600 and 3700 cm^−1^ to characterize the presence of Mg–O–H Ag1 peaks of brucite (Mg(OH)_2_) structure.

Cross-sections of samples were also analyzed by Scanning Electron Microscopy (SEM) with an FEI Quanta 200 environmental scanning electron microscope (Hillsboro, OR, USA) using a back-scattered electron detector (BSED) or a secondary electron detector (SED) in vacuum conditions with an acceleration voltage of 5 kV to avoid a degradation of the material at the Institut de Chimie Séparative (Marcoule, France). Energy-dispersive X-ray spectroscopy (EDX) elemental mapping was performed during 20 min with an energy of 1 keV. The average thickness of CP layers was measured from SEM images on the whole length of the cross-section using the Fiji software to take into account the local variation of the CP layer thickness.

Thin foils for electron microscopy were prepared with a dual-beam FIB using FEI Helios 600,192 NanoLab at the Centre Pluridisciplinaire de Microscopie Electronique et de Microanalyse (Marseille, France). Analyses by Scanning Transmission Electron Microscopy (STEM) were carried out using a JEM ARM 200F (JEOL) transmission electron microscope equipped with a cold-field emission gun and a CEOS aberration corrector of the objective lens [25] at the Laboratoire des Matériaux et Phénomènes Quantiques (Paris, France). Careful attention was paid to the choice of the observation conditions in order to limit sample damage. Indeed, oxide and hydroxide can be damaged by the electron beam at high voltage [26,27]. Thus, we set the TEM voltage to 80 kV. Sample imaging and spatially resolved elemental analysis at the nanoscale were undertaken in scanning TEM mode using high Annular Angle Dark Field (HAADF) imaging and EDX spectroscopy, respectively. A spot size XC was used with a half convergence angle of 16 mrad for both imaging and spectroscopy. HAADF-STEM images were acquired with inner and outer collection angles of 90 and 370 mrad, respectively. EDX spectra were collected on 300 × 300 nm² area during 45 s with an EX-24063 JGT detector. Spatial distributions of elements (Si, Al, Mg, O, F, Na) were determined by integrating the peak area after a background subtraction. The porosity and the mean pore size of the CP layers were extracted from STEM images using binary images with the “trainable WEKA segmentation” and “analyse particles” plug-ins of the Fiji software (version 2). The analysis was performed on 2 different areas of the CP layer and repeated 4 times to reduce the random user error. The protocol of image processing is presented in Supplementary Materials.

## 3. Results

### 3.1. Nature of CP

#### 3.1.1. In Situ GI-XRD

**Figure 2:**
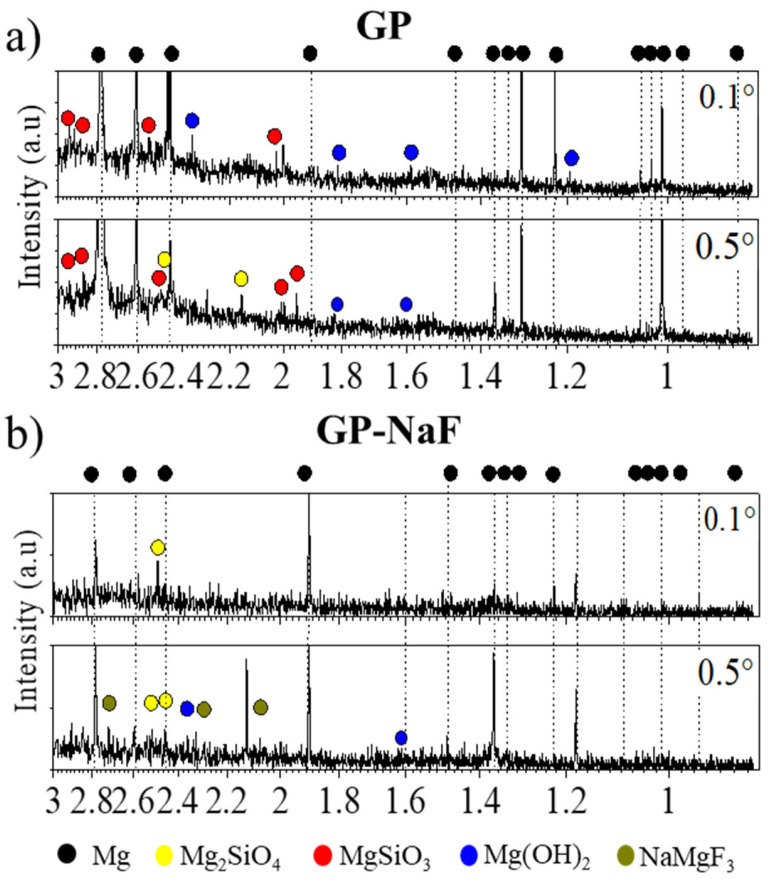
Grazing Incidence hard X-ray Diffraction (GI-XRD) patterns obtained at different incidences angles α_i_ = 0.1 and 0.5° of (**a**) GP and (**b**) GP-NaF.

The GI-XRD patterns at α_i_ = 0.1 and 0.5° of GP and GP-NaF are presented in Figure 2a,b.

As is visible by the GI-XRD patterns, some intense peaks corresponding to the Mg hexagonal phase (ICDD-JCPDS card No. 01-082-9643) are visible but some peak extinctions and/or peak intensity variation with α_i_ can be observed. As examples, the peak at 1.90 Å is not present for the sample GP (Figure 2a) and the peak at 1.46 Å for the sample GP-NaF increases with α_i_. These results can be explained by, first, the existence of various crystallographic grain orientations within the MgZr alloy as shown by the patterns obtained from XRD of several MgZr substrates performed with various orientations (Figure S2). This can be caused by several phenomena: (i) the inhomogenous inclusions of Zr within the alloy [28], (ii) the Zr inclusions limiting the grains growth during the manufacturing process [29], and/or (iii) the preparation of the alloy bare including extrusion and drawing steps. Second, the substrate preparation, cutting and polishing, can induce twining formation [30]. This leads to the formation of new crystalline orientations.

Low intensity peaks attributed to several CP can also be observed. This low intensity may be due to a low amount and/or crystallinity of CP and also by the rough interface existing between MgZr and CP layer. Whatever the samples, without or with NaF, GI-XRD patterns present some peaks which may be attributed to magnesium silicates such as MgSiO_3_ (orhtoenstatite, ruff No.R040093) and Mg_2_SiO_4_ (Forsterite, ruff No. R040057). Mg(OH)_2_ (Brucite, ICDD-JCPDS card No. 01-071-5972) is also observed but mostly at large incidence (i.e., in deeper in the sample). Patterns of the GP-NaF sample show supplementary peaks of NaMgF_3_ (Neighborite, ICDD-JCPDS card No. 01-070-3874).

#### 3.1.2. Raman Microspectroscopy

**Figure 3:**
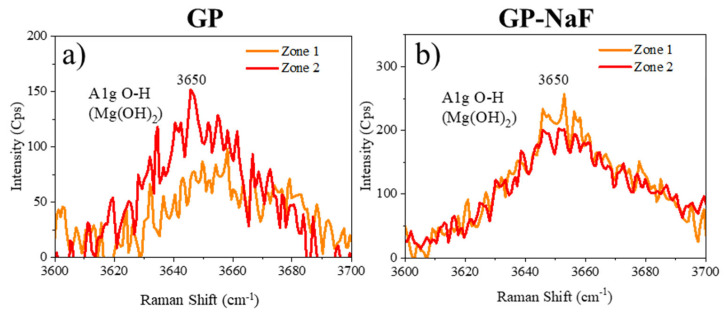
Raman spectra of 2 zones of the corrosion product (CP) layers of samples (**a**) GP and (**b**) GP-NaF.

Local analyses presented in Figure 3 were performed on two zones of the CP layers for each sample.

GP and GP-NaF spectra presented in Figure 3 confirm the presence of Brucite highlighted by GI-XRD, showing a broad peak at 3650 cm^−1^ corresponding to the A1_g_O-H stretching mode in Mg(OH)_2_ crystals [31]. The large peak width attests of a low crystallinity of Mg(OH)_2_.

### 3.2. Morphology and Composition of the Interfaces

**Figure 4:**
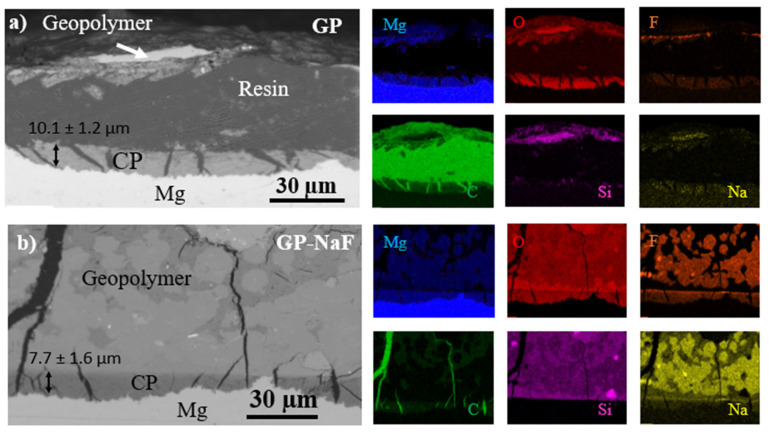
BSED-SEM images and EDX cartographies of Mg, O, F, C, Si, and Na of (**a**) GP and (**b**) GP-NaF.

Images of the sample cross-sections and their relative EDX elemental mapping obtained from SEM-EDX are presented in Figure 4.

These results highlight the presence of cracks perpendicular to the surface of the MgZr substrate in the CP layers and geopolymers for all samples. For GP (Figure 4a), a delamination of the geopolymer from the CP film is noticeable. Indeed, during the geopolymer ageing, cracks and delamination can be formed due to the shrinkage of the geopolymer occurring during the geopolymerization [19] and to a lesser extent during the sample preparation (freezing step) [32,33]. Moreover, the production of hydrogen during the MgZr corrosion may lead to a partial rupture of the CP and/or to CP expansion [12].

The average thickness of CP layers, determined as described in Materials and Methods section, is lower for sample with NaF (7.7 ± 1.6 µm) than for sample without NaF (10.1 ± 1.2 µm).

Regarding the composition of the CP layer, the EDX mapping presented in Figure 4 reveals the presence of Mg, O, and F for all samples. For a sample without NaF, the composition of the CP layer is homogenous. A slight amount of F is detected due to the F impurity inside the poral solution [2] which diffuses through the CP layer, as it has been reported from the characterization of CP layers formed during the corrosion of MgZr substrate in poral solution extracted from geopolymer [17]. The CP layer from the GP-NaF sample is not homogenous and shows an enrichment in F and Na at the surface of the CP layer in contact with the geopolymer.

All of these results are consistent with the GI-XRD data, revealing the existence of Mg(OH)_2_ and of magnesium fluoride such as NaMgF_3_ for GP-NaF. However, magnesium silicates are not detected in the CP layer.

**Figure 5:**
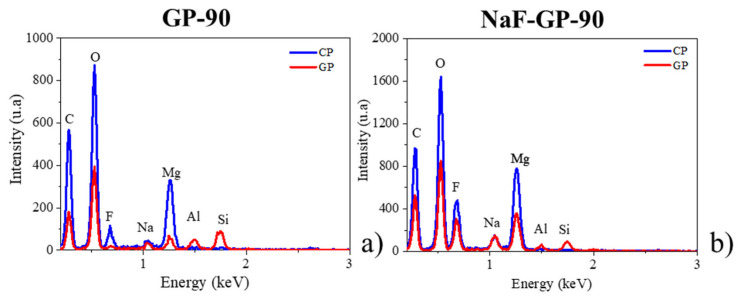
EDX spectra measured in CP at a distance of 5 µm from the CP layer for (**a**) GP and (**b**) GP-NaF samples.

An enrichment of Mg in the geopolymer where Si, O, and Na are detected is also revealed for both samples. The presence of Mg was also confirmed by the local EDX analysis performed at a distance of 5 µm from the CP layer (Figure 5). The diffusion of Mg in the poral solution of the geopolymer during MgZr corrosion and/or the partial dissolution of CP such as Mg(OH)_2_ may explain this enrichment. This would change the composition of the poral solution and lead to the formation of magnesium silicates in the GP zone close to the CP layer as characterized by GI-XRD.

For the sample GP-NaF, some areas are mainly composed of Na and F coming from the precipitation of NaF during the geopolymerization process and/or from the formation of compounds reacting during geopolymerization steps such as Na_2_SiF_6_ or Na_3_AlF_6_ [19]. These species act as a F reservoir that can react with dissolved Mg. The EDX analyses also highlight that F is not homogenously distributed in the geopolymer.

### 3.3. Characterisation at the Nanoscale

#### Morphology and Composition of CP

**Figure 6:**
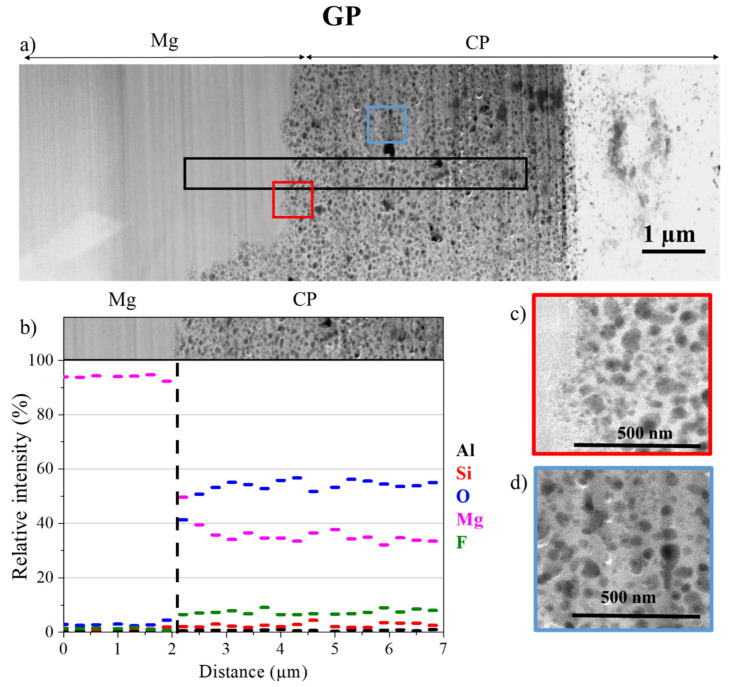
(**a**) Low magnification of HAADF-STEM images assembly of the interface between CP, MgZr for GP thin foil, (**b**) spatial distributions of elements obtained from EDX analysis (black rectangle in (**a**)), zooms of the square areas enclosed in (**c**) red and (**d**) blue in (**a**). The difference in STEM contrast in the CP layer is due to a difference of thin foil thickness.

To study the morphology and the composition of CP layers at a nanoscale, thin foils of the GP and GP-NaF samples were analyzed using STEM-EDX. Figure 6 presents HAADF STEM images of zones located around the MgZr/CP interface and elementary profiles of a GP thin foil and a GP-NaF thin foil, respectively. For the GP sample, the CP layer making up a large part of the thin foil, only part of it has been analyzed.

For the GP sample (Figure 6), the analyzed area of the CP layer is homogenous, porous, and mainly consists of Mg and O with few amounts of F and Si (Figure 5b). The presence of these elements may attests of the formation of Mg(OH)_2−x_F_x_ (x << 1). The Brucite partially substituted by F may present a semi-crystalline structure as characterized by Raman microspectroscopy and GI-XRD.

**Figure 7:**
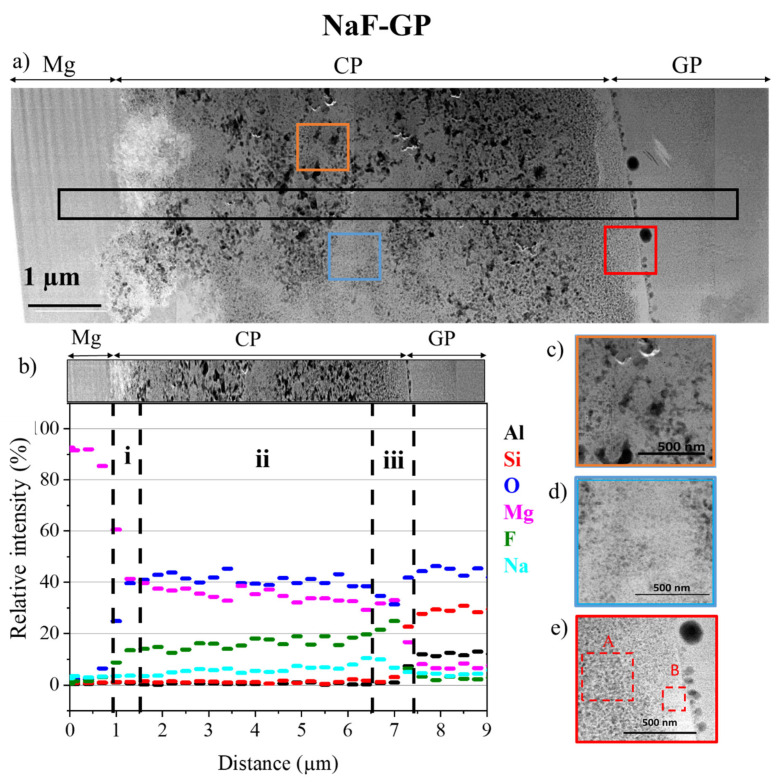
(**a**) Low magnification of HAADF-STEM images assembly of the interface between CP, MgZr for GP-NaF thin foil, (**b**) spatial distributions of elements obtained from EDX analysis (black rectangle in (**a**), zooms of the square areas enclosed in (**c**) red, (**d**) blue, and (**e**) orange in (**a**).

For the GP-NaF sample, the thickness of the CP layer measured from the STEM image of 6.4 µm (Figure 7a) is about the same order of magnitude than the one obtained from SEM image analysis. The CP layer presents several zones highlighted in Figure 7b.

The first zone (i) which is 600 nm-large is located at the surface of MgZr. This zone is dense and mainly consists of Mg, O, and F. This composition may correspond to a mixture of MgZr and Mg(OH)_2−x_F_x_ as reported in [27,34]. The second porous zone (ii) is 5.4 µm large and is a mix of two sets of porous texture. In this zone, the presence of Mg, O, and of a gradient of Na and F may indicate the formation of Mg(OH)_2−x_F_x_ (x ≥ 1) and NaMgF_3_ phases [9]. The two sets of porous texture may be attributed to these two phases as displayed in Figure 7c,d. The last zone of 450 nm close to the GP (iii) is denser than the zone (ii) as attested by the presence of clear area in Figure 7e (zone B) in contact with the CP-GP interface. This goes with an increase of F that may lead to a densification of the CP layer either by the formation of highly substituted Mg(OH)_2−x_F_x_ and the high amount of NaMgF_3_. These results are consistent with GI-XRD analyses.

As observed by SEM-EDX, Mg has diffused into the GP-NaF during the MgZr corrosion. Here too, the magnesium silicates characterized by GI-XRD can be formed into the pores of the geopolymer. Moreover, some holes having the morphology of bubbles are located at the interface between the CP layer and the geopolymer. These “bubbles” may come from the H_2_ release due to the corrosion of MgZr. This shape preservation probably means that high H_2_ production occurs at the first time early after the contact of the MgZr substrate with the geopolymer, i.e., before the end of the geopolymer stiffening (3 to 4 h) [35].

### 3.4. Porosity of CP Layer

**Figure 8:**
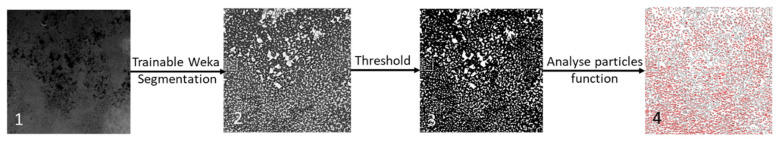
Protocol of image processing for the porous texture analysis of the CP layers.

The porous texture evolution of the CP layers is an important characteristic for the long-term behavior of the waste materials, since it drives the H_2_ diffusion and then its release in the media. Thus, HAADF STEM images of the various zones of the CP layers were treated with the following procedure illustrated in Figure 8 using the Fiji software. First, the scale bar from the raw image was linked to the pixel of the image. Second, after a smoothing process on image 1, Trainable Weka Segmentation was applied to generate image 2 by selecting two zones: one for pores (holes) and one for solid. Third, binary image 3 was then obtained by applying a threshold to image 2. Finally, porosity, pore size distributions, the number of particles analyzed, and their size in µm² were obtained from image 3 using the “Analyse Particles” function. To reduce the user error, this protocol was applied four times on each image. In addition, two different images were taken on each sample to have a better statistic.

**Figure 9:**
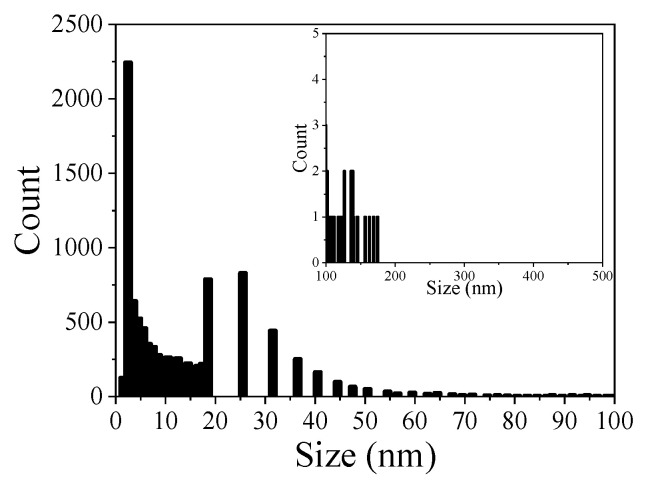
Histogram of the disk size distribution of GP.

**Figure 10:**
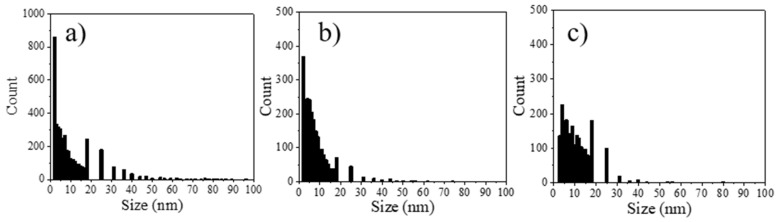
Histograms of the disk size distributions of GP-NaF for (**a**) image (c), (**b**) image (d), and (**c**) image (e) from Figure 7.

The dataset obtained from the image analysis with the protocol presented previously was used to perform statistical analysis of pore size distribution considering that images of the pores have disk shapes. Figure 9 and Figure 10 present the disk (pore) size distribution of GP and NaF-GP, respectively, and the Table 3 the porosity, the mean, and the median pore size obtained with this procedure. The totality of the results obtained by statistical analysis of the dataset from image analysis can be found in Supplementary Materials (Table S1).

The porosity of the CP layers presented in Table 3 are between 40% and 60% lower than the one reported for Mg(OH)_2_ in the literature [34,36]. Moreover, these results show that the CP porosity and the pore size in the GP-NaF sample are smaller than those in the GP sample. The analysis of the CP pore size distributions reveal that more than 75% of the CP pores are smaller than 14.4 nm for the GP-NaF sample and 17.8 nm for the GP sample (Figure 9 and Figure 10).

Additionally, the CP porosity in the GP-NaF sample decreases closer to the CP-GP interface. Moreover, zones (ii) and (iii) present a mix of porous texture. The presence of porous texture (28.9% and 22.5% for zone (ii), and 21.2% porosity and dense solid for zone (iii)) may be associated to two phases, Mg(OH)_2−x_F_x_ and NaMgF_3_, and/or to a non-uniform densification of Mg(OH)_2−x_F_x_ due to the isomorphic substitution of OH by F [21,37].

## 4. Discussion

**Figure 11:**
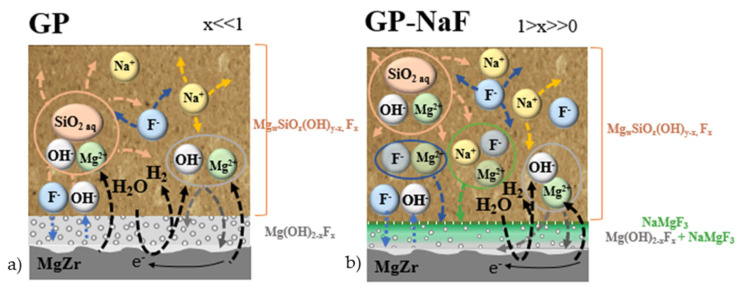
Diagram summarizing the processes which can possibly occur during 3 months of MgZr alloy corrosion in (**a**) GP and (**b**) GP-NaF.

The multiscale characterizations of the CP layers formed in geopolymer without and with NaF have shown the formation of various CP layers having different morphology and composition. The processes at the origin of the formation of these CP are summarized in Figure 11.

### 4.1. General Description of the Samples

In all samples, the CP layer mainly consists of an amorphous/lowly crystalline Mg(OH)_2_ structure. Brucite formation is classically characterized in solution at pH > 10.5 [38] after the local dissolution of magnesium following Equation (1).

Mg^2+^ + 2 OH^−^ → Mg(OH), (1)

because F^−^ is present in poral solution of the geopolymer, the Brucite is partially substituted by an isomorphic exchange of the OH groups by F forming Mg(OH)_2−x,_F_x_ (2).

Mg(OH)_2_ + xF^−^ → Mg(OH)_2−x,_F_x_ + xOH^−^, (2)

with x << 1 when the MgZr corrosion occurred in GP and x >> 0 in GP-NaF.

For the CP layer formed in GP-NaF, NaMgF_3_ is also present and its concentration increases with the distance from the MgZr/CP interface. The presence of NaMgF_3_ in the CP layer can be explained by the high concentration of F^−^ in the poral solution of the geopolymer in contact with the MgZr alloy. This high concentration allows the complete substitution of OH groups by F leading to the formation of MgF_2_, and then, the precipitation of NaMgF_3_. This phenomenon has been already characterized with nanospheres of MgF_2_ reacting with NaF at high concentration (3) to form NaMgF_3_ [39].

MgF_2_ + NaF → NaMgF_3_. (3)

For both samples, magnesium silicates such as Mg_w_SiO_z_(OH)_y-x_F_x_ (with x << 1 in GP and x >> 0 in NaF-GP) are not observed in the CP layer, and thus, are probably localized within the geopolymers. This result is different from the one obtained during the MgZr corrosion in poral solutions extracted from the geopolymers used in this study [17] but probably similar to the ones obtained in the work of Barros et al., as they have detected the presence of magnesium within the geopolymer [21]. To form magnesium silicates, dissolved magnesium diffuses through the CP layer to the poral solution of the geopolymer, reacts with silicates species present in solution, and precipitates. Such a phenomenon particularly favored in nanoconfinement can lead to a pore clogging already observed in model systems [40,41], during glass alteration [42,43,44], but also in geopolymer during ageing time [2]. This may limit the transport of the poral solution to the surfaces of the CP and MgZr.

### 4.2. Origin of the Porous Texture

Depending on the presence of NaF within the geopolymer, the porosity and the pore size distribution of the CP layers are different. Two phenomena can explain these various porous textures.

First, the substitution of OH groups of Brucite by F decreases the cell volume of Mg(OH)_2−x_F_x_, as it is reported in the humite system (from the MgO-SiO_2_-H_2_O compounds) [37] and may lead to a possible densification of the Mg(OH)_2−x,_F_x_ when x increases [12]. Thus, an increase of the F concentration in the media should go with an increase of the Mg(OH)_2−x_F_x_ densification which is the case of the CP layers formed in the GP-NaF sample. Moreover, several studies have showed that an increase of x allows a better stability of Mg(OH)_2−x,_F_x_ in solution regarding its dissolution [45,46].

Second, when F is present within the geopolymer, the formation of two phases within the CP layer may also decrease the porosity. Indeed, when Brucite is formed at the interface MgZr/CP layer, Na and F may diffuse within the porosity, forming Mg(OH)_2−x,_F_x_ and then NaMgF_3_ following (1) and (3). Moreover, the high amount of NaMgF_3_ in the top zone of the CP layer of the GP−NaF sample is associated with a low porosity. This means that NaMgF_3_ precipitation may limit the transport of poral solution and Mg^2+^.

### 4.3. Relation between the Porous Texture and the Protective Properties of the CP Layer

The results obtained in this study have shown that the CP layers formed in GP-NaF is thinner than the CP layer formed in GP sample, 7.7 ± 1.6 µm vs. 10.1 ± 1.2 µm. In these experiments, it is not possible to rigorously compare the MgZr corrosion from the CP layer thickness since the CP formation is not isovolumic regarding MgZr corrosion. The volume occupied by the phases depends mainly on their crystalline structures and CP can undergo dissolution/precipitation processes.

However, taking into account, both the porous texture and the thickness, it is possible to assess the protective properties of the CP layers regarding MgZr corrosion. The CP layer formed in GP-NaF presents a lower porosity and thickness than the CP layer formed in GP, and thus it is probably more protective. These protective properties are associated with, first, the porous texture driving the solution and Mg^2+^ transport through the CP layer and, second, with the phases stability regarding their dissolution. NaF in solution allows the densification of the CP layer by the increase of x in Mg(OH)_2−x,_F_x_ and the formation of NaMgF_3_ and the enhancement of the stability of Mg(OH)_2−x,_F_x_ in solution [45,46]. These results are also in good agreement with several works performed by electrochemical analysis in a similar system [18,19,20] adding NaF as corrosion inhibitor inside a geopolymer.

### 4.4. Comparison with the CP Layers Formed in Poral Solution Extracted from Geopolymer

In our previous study [17], we investigated the evolution of the CP layers formed during the corrosion of MgZr in poral solutions extracted from the same geopolymers with and without NaF used in the present study. Comparing those results, two main differences between the CP layers formed in poral solution and within geopolymer can be noted.

First, in poral solutions, magnesium silicates form within the CP layer, while in geopolymer, the magnesium silicates precipitation occurs mainly within the geopolymer pore network. This highlights that dissolved silicates diffused probably more slowly than F and Na through the geopolymer and to the CP layer. The size of the silicate species, the low solubility of the magnesium silicates, and/or their interactions with CP pore surface may be at the origin of this precipitation within the pores of the geopolymer. Such precipitation may lead to a pore clogging.

Second, with or without corrosion inhibitor, the size of the CP layers formed in poral solutions are lower than the one formed within the geopolymers. Once again, even if the MgZr corrosion cannot be quantified, it is possible to assume that MgZr corrosion is higher in geopolymer than in poral solution. This corrosion difference is mainly due to the amount and the availability of dissolved species present in solution in contact with the MgZr alloy surface that drives the nature of CP formed and probably their protective properties. In corrosion experiments performed with poral solutions, the numerous solution renewals allow the supplying of SiO_2_ (aq) and F^−^ species participating to the formation of a protective CP layer consisting of magnesium silicates and fluorine phases (Mg(OH)_2−x,_F_x_ and NaMgF_3_). In the geopolymer, these dissolved species are not directly available on the total surface of MgZr as illustrated by the F and Na distributions in geopolymer presented on SEM-EDX cartographies in Figure 4. This may lead to the formation of less protective CP layers.

The transport and the discharge of hydrogen formed during MgZr corrosion in geopolymer is different from the one in the solution. In the solution, hydrogen can be released in the atmosphere above the solution while in geopolymer, its production can lead to a repulsion of the poral solution. This may result in a decrease of the MgZr corrosion and in phase precipitation within the pores of the geopolymer.

### 4.5. Proposition of Interconnected Processes

**Figure 12:**
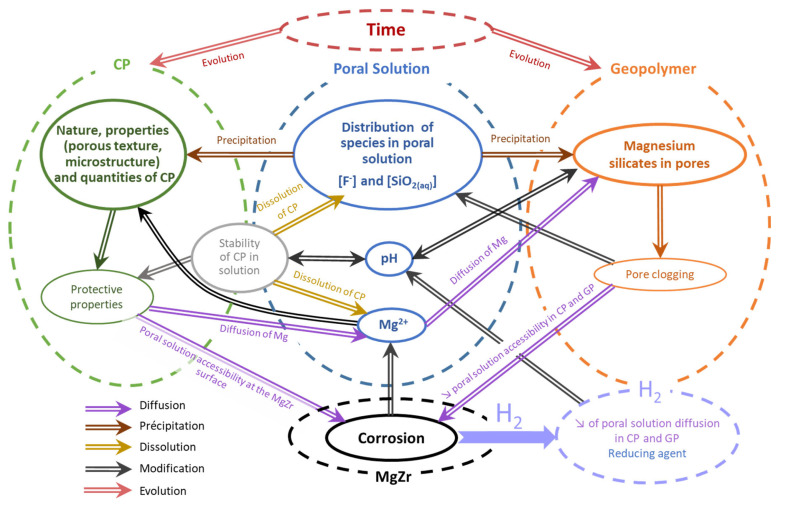
Proposition of interconnected processes involving during the corrosion of MgZr within geopolymers.

Combining the results of this study with those obtained previously from the study of the MgZr corrosion in poral solution [17], a diagram taking into account the processes and their interconnections involving during the corrosion of MgZr alloy within geopolymer is proposed in Figure 12.

When the MgZr alloy is embedded in a geopolymer, the poral solution in contact with the MgZr surface corrodes the alloy that leads to the formation of Mg^2+^ and H_2_. At the pH of the poral solution, dissolved magnesium reacts to form CP such as Mg(OH)_2−x,_F_x_ and NaMgF_3_ when the F^−^ amount is high enough within the media. The reactive diffusion of Mg^2+^ to the poral solution of the geopolymer leads to magnesium silicates precipitation within the pores and may lead to a pore clogging. The formation of protective CP layer that increases with F concentration in poral solution, and magnesium silicates limits the access of the poral solution to the MgZr surface. This process is self-limited in time since the Mg^2+^ supply decreases with the increase of the CP layer protective properties and magnesium silicates precipitation. The consequences of these phenomena, CP formation, magnesium silicates precipitation, and H_2_ formation, may modify the original pH. This modification can change the stability of the CP and leads to their dissolution and then a decrease of their protective properties and a possible resumption of MgZr corrosion.

The last important parameter affecting the evolution of this system is the time. Indeed, CP, geopolymers, magnesium silicates are metastable phases, at least the ones characterized here. With time, it is expected that they become more crystallized. Such crystallization will change their porous texture which will have consequences on the protective properties of the CP layer, the reactive diffusion of the poral solution within the geopolymer, and the H_2_ release in geological disposal.

## 5. Conclusions

In this study, combining in situ and ex situ characterizations and a multiscale approach, we have analyzed the composition, the morphology, and the porous texture of the corrosion products and evidenced the effect of the corrosion inhibitor NaF during the corrosion of MgZr embedded in geopolymer. We determined the formation of Mg(OH)_2−x_F_x_, x increasing with the amount of F species within the geopolymer. In addition, in presence of NaF, NaMgF_3_ forms leading to a decrease of the thickness and the porosity of the corrosion products layer. Moreover, the precipitation of magnesium silicates within the porosity of the geopolymer was highlighted. Finally, combining the results of our previous study, we have proposed a set of interconnected processes occurring during the MgZr corrosion in geopolymer.

Even if these processes are well identified, two factors which can modify them have to be studied in perspective. The first one is the effect of the irradiation due to the radionuclides traces remaining at the surface of the fuel cladding after the spent nuclear fuel removal. Indeed, the irradiation occurring during the corrosion as well as their effect on the geopolymer matrix may modify the evolution of the system. Today, several experiments are ongoing to precisely characterize this effect. The second one is the effect of long timescale. The prediction of the long-term corrosion of MgZr alloy is required to be able to calculate the amount of hydrogen release in the geological disposal. This calculation can only be determined using modelling since the available experiments are performed at a laboratory time scale. Such predictive modelling can be enhanced by the study of natural and archaeological analogous system.

## Figures and Tables

**Table 1:** Impurities in MgZr (data supplied by Neyco).

Impurities	Al	As	Co	Cr	Cu	Fe	Mn	Ni	Sb	Zn	Cl
Quantity (ppm)	<10	<20	<10	22	2	9	11	1	<10	31	10

**Table 2:** Geopolymers, pH, and composition of the solutions used in this study.

Geopolymer	pH	[Na] mmol.L^−1^	[Si] mmol.L^−1^	[F] mmol.L^−1^	[Cl] mmol.L^−1^
GP	12.37	418.9 ± 6.5	46.6 ± 15.8	19.0 ± 0.4	27.4 ± 0.5
NaF-GP	12.40	1116.5 ± 237.8	32.9 ± 9.1	1855.9 ± 37.1	20.0 ± 0.4

**Table 3:** Porosity, mean, and median pore size in CP layers of samples obtained by STEM images analysis using the Fiji software.

Sample	Zone	Porosity (%)	Mean Pore Size (nm)	Median Pore Size (nm)
**GP** (Figure 6)	CP layer	38.2 ± 5.9	13.6	8.5
**GP-NaF** (Figure 7)	Orange square—image (c) Blue square—image (d) Red square—image (e) zone A zone B	28.9 ± 0.7 22.5 ± 2.5 21.2 ± 1.8 Not measureable	10.5 7.9 10.8 Not measurable	6.5 6.1 9.7 Not measurable

## Data Availability

Not applicable.

## Supplementary Materials

The following are available online at https://www.mdpi.com/article/10.3390/ma14082017/s1, Figure S1: Penetration depth of the X-ray beam at 27 keV at a function of the incident angles, Figure S2: XRD patterns of two MgZr substrates analyzed in different position. Table S1: Summary of the results obtained by statistical analysis of dataset from image analysis.
