# Supplementary material for: Corrosion Products Formed on MgZr Alloy Embedded in Geopolymer Used as Conditioning Matrix for Nuclear Waste—A Proposition of Interconnected Processes
Source: Materials (Basel). 2021 Apr 16;14(8):2017. doi: 10.3390/ma14082017 (PMC8073049; doi:10.3390/ma14082017)
Supplement: Supplementary file 1 [file materials-14-02017-s001.zip › materials-1164820-supplementary.pdf]

materials

MDPI

Article

Corrosion Products Formed on MgZr Alloy Embedded in Geopolymer Used as Conditioning Matrix for Nuclear Waste—A Proposition of Interconnected Processes

Rémi Boubon [1], Jaysen Nelayah [2], Samuel Tardif [3], Xavier Deschanels [1] and Diane Rébiscoul [1,*]

[1] ICSM, Université Montpellier, CEA, CNRS, ENSCM, Marcoule, 30207 Bagnols-sur-Cèze, France; remi.boubon@cea.fr (R.B.); xavier.deschanels@cea.fr (X.D.)
[2] Laboratoire Matériaux et Phénomènes Quantiques, Université de Paris, CNRS, 75013 Paris, France; jaysen.nelayah@u-paris.fr
[3] Université Grenoble Alpes, CEA, IRIG-MEM, F-38000 Grenoble, France; samuel.tardif@cea.fr
* Correspondence: diane.rebiscoul@cea.fr

Citation: Boubon, R.; Nelayah, J.; Tardif, S.; Deschanels, X.; Rébiscoul, D. Corrosion Products Formed on MgZr Alloy Embedded in Geopolymer Used as Conditioning Matrix for Nuclear Waste—A Proposition of Interconnected Processes. *Materials* **2021**, *14*, 2017. https://doi.org/10.3390/ma14082017

Academic Editor: Herman Potgieter

Received: 15 March 2021
Accepted: 8 April 2021
Published: 16 April 2021

Publisher's Note: MDPI stays neutral with regard to jurisdictional claims in published maps and institutional affiliations.

Copyright: © 2021 by the authors. Submitted for possible open access publication under the terms and conditions of the Creative Commons Attribution (CC BY) license (http://creativecommons.org/licenses/by/4.0/).

Penetration depth of the X-ray beam at 27 keV at a function of the incident angles

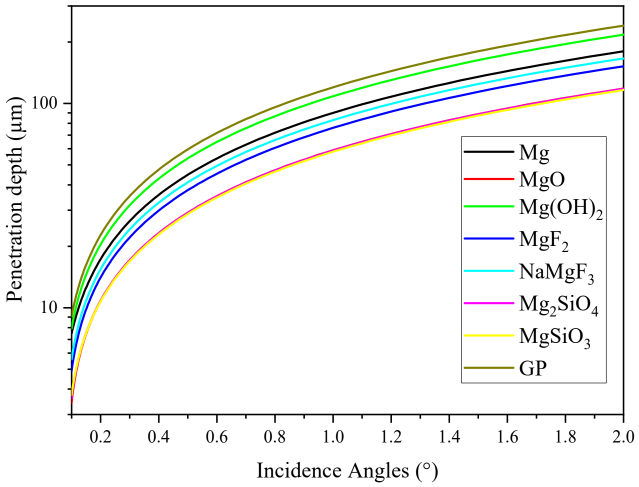

Figure S1. Penetration depth of the X-ray beam at 27 keV at a function of the incident angles.

XRD analyses of two MgZr samples: highlight of various crystallographic grain orientations

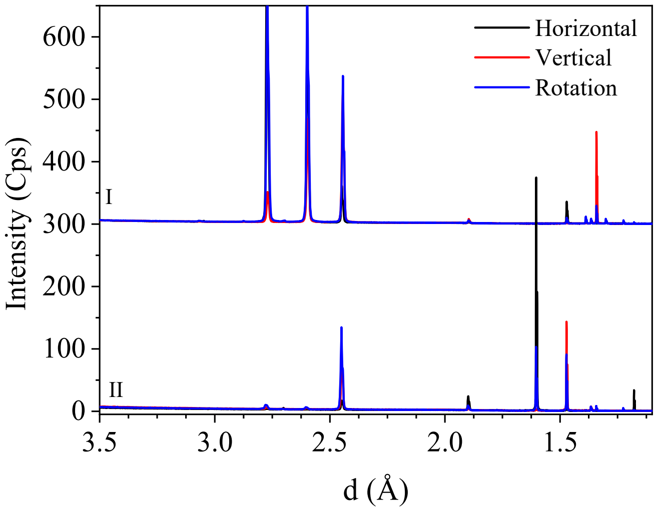

Figure S2. XRD patterns of two MgZr substrates analyzed in different position.

Table S1. Summary of the results obtained by statistical analysis of dataset from image analysis.

	**GP**	**GP-NaF image (c)**	**GP-NaF image (d)**	**GP-NaF image (e)**
N total	9874.00	4103	2352	2183
Mean (nm)	13.63	10.50	7.82	10.80
Standard Deviation (nm)	18.71	13.1	7.56	6.61
Lower 95% CI of Mean (nm)	13.26	10.10	8.09	10.52
Upper 95% CI of Mean (nm)	14.00	10.90	8.09	11.08
Variance	349.99	173.31	44.31	43.8
SD times 2	37.42	26.32	13.31	13.23
SD times 3	56.12	39.49	19.69	19.85
Minimum (nm)	1.22	1.63	1.62	2.58
1st Quartile (Q1) (nm)	2.82	2.81	3.63	5.78
Median (nm)	8.47	6.50	6.08	9.68
3rd Quartile (Q3) (nm)	17.84	13.11	10.01	14.41
Maximum (nm)	822.63	229.89	13.56	79.78

References of Supplementary Data

1. X-Ray Attenuation Length. Available online: http://henke.lbl.gov/optical_constants/atten2.html (accessed on 3 Februray, 2020).
2. Henke, B.L.; Gullikson, E.M.; Davis, J.C. X-ray interactions: Photoabsorption, scattering, transmission, and reflection at E = 50-30,000 eV, Z = 1-92. *At. Data Nucl. Data Tables* **1993**, *54*, 181–342, doi:10.1006/adnd.1993.1013.
